# Light cupula of the horizontal semicircular canal occurring alternately on both sides: a case report

**DOI:** 10.1186/s12901-015-0015-z

**Published:** 2015-03-14

**Authors:** Jung Eun Shin, Chang-Hee Kim

**Affiliations:** Department of Otorhinolaryngology-Head and Neck Surgery, Konkuk University Medical Center, Konkuk University School of Medicine, 120-1 Neungdong-ro (Hwayang-dong), Gwangjin-gu, Seoul 143-729 Republic of Korea

**Keywords:** Direction-changing positional nystagmus, Light cupula, Head-roll test, Positional vertigo

## Abstract

**Background:**

The light cupula is a condition wherein the cupula of the semicircular canal has a lower specific gravity than its surrounding endolymph. It is characterized by a persistent geotropic direction-changing positional nystagmus in the supine head-roll test, and the identification of a null plane with slight head-turning to either side.

**Case presentation:**

This study describes a case of recurring light cupula that occurred alternately on both sides. At the first episode, a null plane was identified on the right side, which led to the diagnosis of a light cupula on the right side. At the second episode, a null plane was identified on the left side, leading to the diagnosis of a light cupula on the left side.

**Conclusion:**

This is the first case report of recurring light cupula alternately involving both sides. Although the pathophysiology is not entirely understood yet, the light cupula should be considered as one of causes of recurrent positional vertigo.

**Electronic supplementary material:**

The online version of this article (doi:10.1186/s12901-015-0015-z) contains supplementary material, which is available to authorized users.

## Background

The condition of “light cupula”, characterized by a cupula with a lower specific gravity than its surrounding endolymph, has been introduced as an emerging concept accounting for positional vertigo [1-4]. In cases where the light cupula involves the horizontal semicircular canal (hSCC), a persistent geotropic direction-changing positional nystagmus (DCPN) without latency or fatigability is typically observed in the supine head-roll test. A null plane, in which the nystagmus disappears, can be identified when the patient’s head is slightly turned to the right or left side while the patient is in the supine position. In this report, we will describe a case of recurrent light cupula occurring alternately on both sides with an interval of 2 months.

## Case presentation

**Figure 1:**
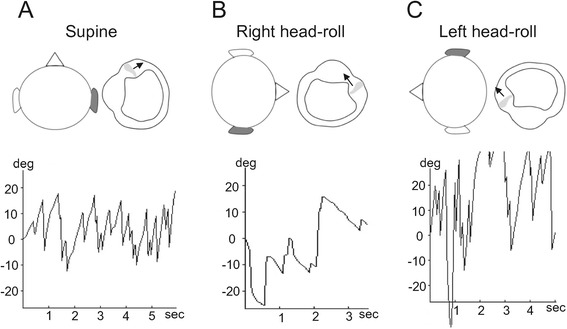
**The instance of light cupula observed in the right hSCC.** The patient’s head and right hSCC were viewed from the top of the patient’s head. **(A)** In the supine position, a left-beating nystagmus (maximal SPV = 33°/s) was persistently observed due to an utriculofugal deflection of the cupula. **(B)** When the patient’s head was turned to the right while in the supine position, the right hSCC was activated because of an utriculopetal deflection of the cupula resulting in a persistent right-beating nystagmus (maximal SPV = 10°/s). **(C)** The cupula of the right hSCC was deflected utriculofugally when the head was turned to the left side, which caused a persistent left-beating nystagmus (maximal SPV = 53°/s). Note that the intensity of the nystagmus is stronger in left-head rolling than in right head-rolling. hSCC, horizontal semicircular canal; SPV, slow-phase velocity.

A previously healthy 38-year-old man who suddenly developed positional vertigo was referred to our hospital. He reported that he woke up early in the morning because he felt severe vertigo. Vertigo symptom persisted even when the patient’s head was kept still, which was aggravated with a position change. He did not complain of audiological symptoms or headaches, and reported no previous history of vertigo or neurological disorders including migraine. The otoscopic examination revealed a normal tympanic membrane. Further examination yielded a very weak left-beating spontaneous nystagmus when the patient was in the sitting position, and the head impulse test revealed no catch-up saccade. Additionally, there were no focal neurological deficits found. Examinations of pure tone audiometry revealed normal hearing on both sides. The patient’s eye movement was examined at various head positions and was recorded using a goggle equipped with an infrared camera (SLMED, Seoul, Korea), thus facilitating the “light cupula” diagnosis. A right-beating nystagmus was observed when the patient bowed his head at 90°. While in the supine position, a horizontal nystagmus beating toward the left side was persistently observed, and the maximal slow-phase velocity (SPV) was 33°/s (Figure 1A, Additional file 1). When the patient’s head was turned to the right (Figure 1B, Additional file 2) or left (Figure 1C, Additional file 3) at 90° in the supine position, a geotropic DCPN continued persistently as long as the position was maintained. The intensity of the nystagmus was stronger with head-roll to the left (maximal SPV = 53°/s) than to the right (maximal SPV = 10°/s). A null plane, at which the nystagmus ceases and the direction of nystagmus changes, was identified when the patient turned his head slightly to the right (25 ~ 30°) while in the supine position, leading to a diagnosis of a light cupula on the right side. The diagnostic criteria for the light cupula are the presence of a persistent geotropic DCPN on the supine head-roll test and the identification of a null plane [1]. Magnetic resonance imaging of the brain revealed no abnormal finding.

The patient, after sharing his perspective on the treatment, was prescribed vestibular suppressants for symptomatic relief for one week, and the positional vertigo and nystagmus disappeared within 1 week without adverse effect of the drugs.

**Figure 2:**
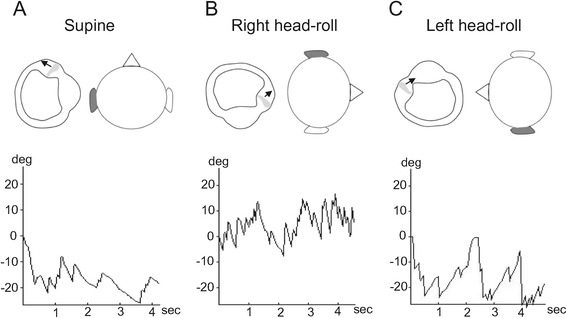
**The instance of light cupula observed in the left hSCC.** The patient’s head and the magnified left hSCC were viewed from the top of the patient’s head. **(A)** In the supine position, a left-beating nystagmus (maximal SPV = 19°/s) was persistently observed due to an utriculofugal deflection of the cupula. **(B)** When the patient’s head was turned to the right side, the left hSCC was inhibited because of the utriculofugal deflection of the cupula resulting in a persistent right-beating nystagmus (maximal SPV = 38°/s). **(C)** When the head was turned to the left side, the cupula of the left hSCC was deflected utriculopetally, which caused a persistent left-beating nystagmus (maximal SPV = 10°/s). Note that the intensity of the nystagmus is stronger in right-head rolling than in left head-rolling. hSCC, horizontal semicircular canal; SPV, slow-phase velocity.

Two months later, the patient revisited our clinic with complaints of severe positional vertigo akin to the previously experienced episode of vertigo. Thorough neurological examinations revealed no focal neurologic signs, and the patient’s hearing was normal on both sides. The patient denied alcohol intake within a week before the vertigo attack. A weak spontaneous nystagmus beating toward the right side was observed when the patient was in the sitting position, and a weak left-beating nystagmus was observed when the patient bowed his head at 90°. In the supine position, a right-beating nystagmus was continuously observed, and the maximal SPV was 19°/s (Figure 2A, Additional file 4). When the patient’s head was turned to the right (Figure 2B, Additional file 5) or left (Figure 2C, Additional file 6) at 90° in the supine position, a persistent geotropic DCPN was observed. The intensity of the nystagmus was stronger with a head-roll to the right (maximal SPV = 38°/s) than to the left (maximal SPV = 10°/s). A null plane was identified when the patient turned his head slightly to the left (25 ~ 30°) in the supine position, which led to the diagnosis of a light cupula on the left side.

## Discussion

Our patient suffered from recurring positional vertigo, which is, as far as we know, the first report of recurring light cupula alternately involving both sides. There are two points that may be addressed from this observation: (1) the orientation of the hSCC cupular axis with regard to the gravitational vector, (2) the pathophysiology of the light cupula.

The pathophysiology of light cupula is still unclear. The attachment of light debris to the cupula has been suggested as a cause of light cupula [2], but the light debris has not been identified yet. Others have proposed that the increase in the specific gravity of the endolymph may contribute to light cupula [1,5], which was further supported by recent findings suggesting that light cupula can be accompanied by sudden sensorineural hearing loss ipsilaterally [6], and that the condition of light cupula may involve all 3 SCCs on the same side [7]. The most interesting finding of this case report is that the condition of light cupula occurred on both sides, of which the mechanism is still obscure. If relative specific gravity of the endolymph to the cupula is changed by systemic influence such as hormonal imbalance, or both labyrinths are alternately affected by inner ear ischemia, bilateral involvement of the light cupula may be possible.

In previous studies, the cupular axis of hSCC has been described as running medial to lateral in direction, and the angle between the sagittal plane and the cupular axis was variable, ranging from 11° to 58° [1,2,5,8]. In this condition, the side of the null plane corresponds to the side of light cupula, and is suggested to be the most important finding for the determination of the affected side [1]. At the first episode of vertigo, which was caused by a light cupula in the right hSCC, a null plane was identified on the right side, and the nystagmus was stronger in the left head-roll than in the right. A right-beating nystagmus was observed in the bowing position, and a left-beating nystagmus was observed when the patient was placed in the supine position. At the second episode, persistent geotropic DCPN was more intense in the right head-roll than in the left, and a null plane was identified on the left side. At both episodes of vertigo, the intensity of nystagmus during head-roll test was greater when the head was turned to the healthy side than the lesioned side, which ran counter to Ewald’s second law. Although further investigation is needed to explain this, possible mechanisms can be speculated as follows; (1) incomplete head rotation to the lesioned side in supine head-roll test, (2) anatomical variations of hSCC within the temporal bone such as excessive upward-tilting of anterior end of the hSCC, (3) the influence of otolith organ inputs upon hSCC ocular reflex by way of the velocity storage integrator [9], and (4) anatomical variation of the cupula within hSCC in a way that the axis of cupula is running lateral to medial in direction.

Because the incidence of light cupula in patients showing geotropic DCPN was as high as 14% [1], meticulous investigation of the duration and latency of positional nystagmus during supine head-roll test is essential for differential diagnosis between light cupula and hSCC canalolithiasis.

## Conclusion

This is the first case report of recurring light cupula alternately involving both sides, which should be considered in the differential diagnosis of recurrent positional vertigo.

### Consent

Written informed consent was obtained from the patient for publication of this Case report and any accompanying images.

## Additional files

Additional file 1:
**Nystagmus in the supine position at the first vertigo attack.** Left-beating nystagmus is observed. Additional file 2:
**Head-roll to the right at the first vertigo attack.** Right-beating nystagmus is observed. Additional file 3:
**Head-roll to the left at the first vertigo attack.** Left-beating nystagmus is observed. Additional file 4:
**Nystagmus in the supine position at the second vertigo attack.** Right-beating nystagmus is observed. Additional file 5:
**Head-roll to the right at the second vertigo attack.** Right-beating nystagmus is observed. Additional file 6:
**Head-roll to the left at the second vertigo attack.** Left-beating nystagmus is observed.

## Abbreviations

DCPN: Direction-changing positional nystagmus
hSCC: Horizontal semicircular canal
SPV: Slow-phase velocity

## References

[1] Kim CH, Kim MB, Ban JH (2014). Persistent geotropic direction-changing positional nystagmus with a null plane: the light cupula. Laryngoscope.

[2] Ichijo H (2012). Persistent direction-changing geotropic positional nystagmus. Eur Arch Otorhinolaryngol.

[3] Bergenius J, Tomanovic T (2006). Persistent geotropic nystagmus–a different kind of cupular pathology and its localizing signs. Acta Otolaryngol.

[4] Hiruma K, Numata T (2004). Positional nystagmus showing neutral points. ORL J Otorhinolaryngol Relat Spec.

[5] Hiruma K, Numata T, Mitsuhashi T, Tomemori T, Watanabe R, Okamoto Y (2011). Two types of direction-changing positional nystagmus with neutral points. Auris Nasus Larynx.

[6] Kim CH, Choi JM, Jung HV, Park HJ, Shin JE (2014). Sudden sensorineural hearing loss with simultaneous positional vertigo showing persistent geotropic direction-changing positional nystagmus. Otol Neurotol.

[7] Kim CH, Shin JE, Shin DH, Kim YW, Ban JH (2014). “Light cupula” involving all three semicircular canals: a frequently misdiagnosed disorder. Med Hypotheses.

[8] Bisdorff AR, Debatisse D (2001). Localizing signs in positional vertigo due to lateral canal cupulolithiasis. Neurology.

[9] Ichijo H (2012). Is horizontal semicircular canal ocular reflex influenced by otolith organs input?. Acta Otolaryngol.

